# Noise alters guinea pig’s blood-labyrinth barrier ultrastructure and permeability along with a decrease of cochlear Claudin-5 and Occludin

**DOI:** 10.1186/s12868-014-0136-0

**Published:** 2014-12-24

**Authors:** Yong-Xiang Wu, Guo-Xia Zhu, Xin-Qin Liu, Fei Sun, Ke Zhou, Shuang Wang, Chun-Mei Wang, Jin-Wen Jia, Jian-Tao Song, Lian-Jun Lu

**Affiliations:** Department of Otolaryngology, Head and Neck Surgery, Xijing Hospital, Fourth Military Medical University, Xi’an, Shaanxi China; Department of Otorhinolaryngology, Head and Neck Surgery, NO.474 Hospital of Chinese PLA, Urumchi, Xinjiang China; Department of Otorhinolaryngology, The Affiliated Guangren Hospital of Xi’an Jiaotong University College of Medicine, Xi’an, Shaanxi China; Department of Occupational and Environmental Health, School of Public Health, Fourth Military Medical University, Xi’an, Shaanxi China; Department of Physiology, Preclinical School of Medicine, Fourth Military Medical University, Xi’an, Shaanxi China; Central Laboratory, Preclinical School of Medicine, Fourth Military Medical University, Xi’an, Shaanxi China

**Keywords:** Blood-labyrinth barrier, Noise exposure, Tight-junction proteins

## Abstract

**Background:**

Noise exposure (NE) is a severe modern health hazard that induces hearing impairment. However, the noise-induced ultrastructural changes of blood-labyrinth barrier (BLB) and the potential involvements of tight junction proteins (TJP) remain inconclusive.

We investigated the effects of NE on not only the ultrastructure of cochlea and permeability of BLB but also the expression of TJP within the guinea pig cochlea.

**Results:**

Male albino guinea pigs were exposed to white noise for 4 h or 2 consecutive days (115 dB sound pressure level, 6 hours per day) and the hearing impairments and light microscopic change of BLB were evaluated with auditory brainstem responses (ABR) and the cochlear sensory epithelia surface preparation, respectively. The cochlear ultrastructure and BLB permeability after NE 2d were revealed with transmission electron microscope (TEM) and lanthanum nitrate-tracing techniques, respectively. The potential alterations of TJPs Claudin-5 and Occludin were quantified with immunohistochemistry and western blot. NE induced significant hearing impairment and NE 2d contributed to significant outer hair cell (OHC) loss that is most severe in the first row of outer hair cells. Furthermore, the loosen TJ and an obvious leakage of lanthanum nitrate particles beneath the basal lamina were revealed with TEM. Moreover, a dose-dependent decrease of Claudin-5 and Occludin was observed in the cochlea after NE.

**Conclusions:**

All these findings suggest that both decrease of Claudin-5 and Occludin and increased BLB permeability are involved in the pathologic process of noise-induced hearing impairment; however, the causal relationship and underlying mechanisms should be further investigated.

**Electronic supplementary material:**

The online version of this article (doi:10.1186/s12868-014-0136-0) contains supplementary material, which is available to authorized users.

## Background

Hearing impairment occurs in many noise exposure (NE) environments, including occupational (e.g., industrial, military), or recreational (e.g., musical concerts, hunting) settings and is closely related with the underlying cochlear pathologies including the increased endocytosis, vacuolation, mitochondrial lesion, elevated intracellular Ca^2+^ concentration and the generation of reactive oxygen species that lead to hair cell death [1-3]. Acute exposure to high noise or chronic exposure to intermediate one causes moderate acoustic impairment such as temporary threshold shifts. This moderate impairment is accompanied with reversible physiological changes in hair cells and the blood-labyrinth barrier (BLB), suggesting a reversible impairment nature [4-6].

Among these pathological changes, the alteration of BLB permeability caused by NE has become the research focus because the integrity of BLB plays a crucial role in stabilizing cochlear microenvironment and maintaining the function of inner ear [7,8]. Cochlear disorders including the changes of ionic concentration and osmotic pressure, metabolic disturbances and so on are always accompanied with BLB impairment [6,7]. However, it is still inconclusive whether NE can really impair BLB permeability since there are a few evidences that are either for [9] or against [10] this concept.

We first confirmed that NE did impair BLB permeability and then we tried to reveal the underlying molecular events. Previous studies suggested that Na+/K + −ATPase α1 was abundant in the BLB and is one of the potential mediators for noise-induced permeability impairment [9]. However, this hypothesis can not explain all BLB permeability impairments and there might be other candidates. Tight junctions (TJs) exist extensively in BLB and contribute mainly to the permeability of BLB [11]. Thus, alteration of TJs can significantly affect the BLB permeability.

The core of TJs are composed of Claudin and Occludin which are membrane proteins that express extensively in the blood–brain barrier (BBB) and contribute to the integrity of BBB [12-15]. Claudin and Occludin exist in dimers and interact with the same type of protein on adjacent cells to form “shoelace-like” structures which are connected in a closed chain to seal the intercellular space [16]. Additionally, Claudin and Occludin are connected with cytoplasmic proteins and play a unique barrier function. The intracellular domains of Claudin-5 and Occludin are connected with cytoplasmic adhesion proteins and their extracellular domains interact with other TJ membrane proteins [16].

Previous study has suggested that Occludin is extensively expressed in BLB and its phosphorylation activated by interaction between PKCη and ATP1A1 may contribute to the BLB integrity [9]. However, the alterations of Occludin level in BLB after NE is not reported. On the other hand, although Claudin-5 is another important TJ protein (TJP), till now, there is no report on its expression in BLB or its involvement in maintaining the BLB integrity.

Therefore, we investigated in the current study the ultrastructure and permeability changes of BLB and analyzed how the distribution and expression of Claudin-5 and Occludin in the BLB were altered after NE to offer more evidences for the mechanisms of noise-induced BLB ultrastructure change and hearing impairments.

## Methods

### Experimental animals

One hundred and fourteen healthy male albino guinea pigs (weighing from 250 to 350 g) with a normal Preyer’s reflex were obtained from the animal centre of the Fourth Military Medical University (FMMU) and used in the current study. Each animal had been raised alone with sufficient food and water in a tranquil animal cage for 5 days prior to the test. All experiment procedures were supervised and approved by the Institutional Animal Care and the Committee on Animal Research in FMMU.

### Experiment group and NE

All animals were randomly divided into control (Ctrl, n = 38), 4 h NE (NE 4 h, n = 38) and 2 d NE (NE 2d, n = 38) groups, respectively. Animals in the NE groups were exposed to white noise at 115 dB sound pressure level (SPL) for either 4 h (NE 4 h) or 6 h/day in two consecutive days (NE 2d). In the control group, animals were treated according to the above-mentioned methods except for the white noise exposure.

The NE protocol was described previously in our department [17]. Briefly, NE was conducted in the ventilated soundproof cabinet where the animals had free access to the food and water. A RadioShack Super-tweeter located above the cages generated a noise (white noise, 115 dB SPL) which was then amplified by a power amplifier (Yamaha AX-500U) and delivered to a loudspeaker. The homogeneity of the sound field was confirmed using a sound-level meter (Bruel and Kjaer Type 2606) that was immobilized within the cabinet.

### Auditory brainstem responses (ABR) measurement

ABRs to both click stimuli and pure tone frequencies in a soundproof chamber on the day before NE and at approximately 24 h after the last sound exposure were used to evaluate hearing alterations of guinea pigs from Ctrl, NE 4 h and NE 2d groups. Each animal was gently anesthetized with an intraperitoneal (ip) injection of pentobarbital sodium (30 mg/kg) when the body was kept warm. The reference electrode beneath the pinna of the test ear, the ground one beneath the apex of the nose and the active one beneath the skin on the top of the head were subcutaneously placed on site within 5 min after the anesthesia. ABR test started immediately after needle electrodes implantation. Each test ear received the stimulus signal at a repeating rate of 10/s generated through Intelligent Hearing Systems (Bio-logic Systems and TDT-RZ6, USA) and the stimulus signal was delivered through earphones with a 10 min interval between left and right ears. The click stimuli was delivered to the left and right ears at the 10 min interval. The stimuli intensity was decreased gradually by a 5-dB step until a visually discernible ABR waveform disappeared and the lowest sound level that caused this waveform was defined as ‘threshold’. Five repetitions of each threshold were presented. And meanwhile the waves were amplified ten times by Intelligent Hearing Systems. The highest sound level was no more than 90 dB to avoid drastic acoustic trauma.

### Epithelia surface preparation and outer hair cell (OHC) count

Cochlear sensory epithelia surface preparation and OHC count in animals from Ctrl (n = 4 from 2 animals), NE 4 h (n = 4 from 2 animals) or NE 2d (n = 4 from 2 animals) groups were carried out following a previous study from our department [17]. The animals were perfused transcardially with freshly prepared 4% paraformaldehyde in 0.1 M phosphate-buffered (PB, pH 7.4) under deep anesthesia with pentobarbital sodium (60 mg/kg) and the cochleae were removed immediately. Cochleae were further post-fixed through the open round windows and cochlear apexes with the same fixative for overnight. After removal of the bony capsule, the spiral ligament, stria vascularis and Reissner’s membrane were separated under a dissecting microscope. Each turn of the Corti organ was detached from the bony modiolus. The sensory epithelium was trimmed, and surface preparations were stained for actin using fluoresceinyl-aminomethyldithiolano-phalloidin (catalog no. Alx-350-268-MC01, Enzo Life Sciences, Farmingdale, NY, U.S.A). The sensory epithelia surface structures were carefully examined for missing cells or stereocilia under a fluorescence microscope (Olympus BX-51, Tokyo, Japan). OHCs were counted by hand in five consecutive images from each slide. The principle of counting is that if OHCs are not integrity in the edge of image, the above and the left they lie in are only available. The missing hair cells and stereocilia were quantified along the entire basilar membrane. The percentage of missing OHCs in each row were calculated and compared among the three groups [17].

### Transmission Electron Microscopy (TEM)

Animals from Ctrl or NE 2d group were deeply anesthetized and perfused transcardially with 0.9% saline followed by fixative of 2.5% glutaraldehyde and 4% paraformaldehyde in 0.1 M PB (pH 7.4) immediately after the ABR measurements. The cochleae were removed immediately, the spiral ligament and stria vascularis were removed under a dissecting microscope (OLYMPUS SZX12, Japan). and were divided into small blocks at about 1 mm^3^ size then fixed with 2.5% glutaraldehyde under 4°C. Ultra-thin sections at 70 nm thickness were prepared, and stained with uranyl acetate and lead citrate following the conventional protocol. The material was observed under TEM (JEM-2000EX, Japan) to reveal the ultrastructure of cochlea and BLB.

### Lanthanum nitrate particles tracing and TEM observation

The BLB permeabilities of animals from Ctrl (n = 4 from 2 animals) and NE 2d (n = 4 from 2 animals) groups were examined with lanthanum nitrate-tracing TEM examination according to Hoffestein’s method [18] and the modification by Leeson [19]. After guinea pigs were transcardially perfused with the fresh fixative of lanthanum nitrate and sodium cacodylate, the cochleae were removed and immersed in the same fixative at 4°C for at least 24 h. Then the cochlear lateral wall tissues were dissected from bony structure under the dissecting microscope. Then the tissues were postfixed in 1% osmium, gradually dehydrated in a graded acetone series, embedded in Araldite plastic, sectioned, stained with lead citrate and uranyl acetate. Finally, the specimens were observed under TEM (JEM-2000EX, Japan).

### Immunohistochemistry

Guinea pigs from Ctrl (n = 4 from 2 animals), NE 4 h (n = 4 from 2 animals) or NE 2d (n = 4 from 2 animals) groups were perfused transcardially with freshly prepared 4% paraformaldehyde in 0.1 M PB (pH 7.4) under deep anesthesia with pentobarbital sodium (60 mg/kg). The cochleae were postfixed with the same fixative at 4°C for at least 24 hours, decalcified with 10% EDTA (PBS, pH 7.4) at 23°C for 2 weeks, dehydrated in 30% sucrose for 24 hours, embedded in OCT glue, and sectioned at 10 μm thickness in the mid-modiolus plane on a cryostat.(CM 3000,.Germany) The sections were soaked with 0.3% hydrogen peroxide in methanol for 10 min to inactivate endogenous peroxidase and blocked with goat serum (Jackson Immunoresearch Lab, U.S.) at 37°C for 30 min. Then they were incubated with rabbit anti-Occludin polyclonal antibody (catalog no.sc-5562, Santa Cruz Biotechnology, INC., 1:200) or rabbit anti-Claudin-5 polyclonal antibody (catalog no.sc-28670, Santa Cruz Biotechnology, INC., 1:200) at 4°C overnight. Primary antibodies were omitted from the negative control for both two antibodies. Then, sections were incubated with biotinylated goat anti-rabbit IgG (catalog no.SA1022, Boster, China) at 37°C for 30 min, followed by incubation with streptavidin-biotin peroxidae complex (SABC) (catalog no.SA1022, Boster, China) at 37°C for 30 min and then 3, 3^′^-diaminobenzidine tetrahydrochloride substrate (DAB) (catalog no.D5637, Sigma, U.S.) for coloration. The sections were counterstained with hematoxylin, dehydrated in a graded ethanol series, hyalinized in xylene, mounted on collagen coated glass slides, cover slipped and visualized under Light microscope (Olympus BH-2, Japan). Three washes in 0.01 M PBS were used in the interval of each step.

### Western blot

Ten guinea pigs from each group were sacrificed immediately after the last ABR measurement by decapitation on ice and the whole cochlear lateral walls and retina tissues from the same group were harvested, pooled and stored at −80°C until use, respectively. The pooled tissues were lysed in sample buffer containing 1 × Tris-EDTA, NaCl (100 mM), 1% Triton X-100, 1× protease inhibitors (all components were obtained from Sigma-Aldrich). After mechanical lysis and protein extraction, protein concentration was determined by using a BCA assay kit (Sigma-Aldrich). Equal amounts (about 100 ng/lane) of protein were loaded on 12% sodium dodecyl sulfate-polyacrylamide gels (SDS-PAGE) (90 V, for 80 minutes at room temperature), electrophoresed, and transferred to nitrocellulose membranes by electroblotting (30 V, overnight at 4°C) in 1× transfer buffer (Bio-Rad). After being blocked in 5% nonfat dry milk and 0.1% Tween-20 in PBS (pH 7.4, 0.01 M) for 1 h at 25°C, the membranes were incubated overnight at 4°C with different primary antibodies (rabbit anti-Occludin polyclonal antibody, catalog no.sc-5562, Santa Cruz Biotechnology, Inc., 1:200 or rabbit anti-Claudin-5 polyclonal antibody, catalog no.sc-28670, Santa Cruz Biotechnology, Inc., 1:200). The membranes were then incubated with a HRP-conjugated secondary antibody (goat anti-rabbit anti-body; Bio-Rad) for 1 h at room temperature. Equal protein loading was confirmed by stripping the blots and reprobing them with a polyclonal rabbit anti-GAPDH antibody (Chemicon International, Temecula, CA, 1:5000) followed by incubation with the same HRP-conjugated secondary antibody (goat anti-rabbit anti-body; Bio-Rad). The protein bands were detected by chemiluminescence detection technique (FluorChem FC2, Alpha Innotech) and the gray density of detected bands were analyzed with NIH ImageJ software.

### Statistic analysis

The descriptive analysis was used for cochlear ultrastructure and BLB permeability data. The data for OHC loss, ABR, IHC and western blot experiments were expressed as mean ± standard error mean (SEM). One-way analysis of variance (ANOVA) with Turkey HSD *post hoc* test or two-way analysis of variance was performed with SPSS version 13.0 software package (SPSS Inc., Chicago, Ill.) to detect the difference of OHC loss, ABR shift, ABR threshold, IHC and western blot data. The significant difference was considered at P < 0.05 level.

## Results

### NE impairs hearing and the cochlea hair cells

White noise was delivered in NE 4 h or NE 2d group and the morphological and functional impairments of cochleae were evaluated with the OHC count and ABR measurement, respectively.

**Figure 1:**
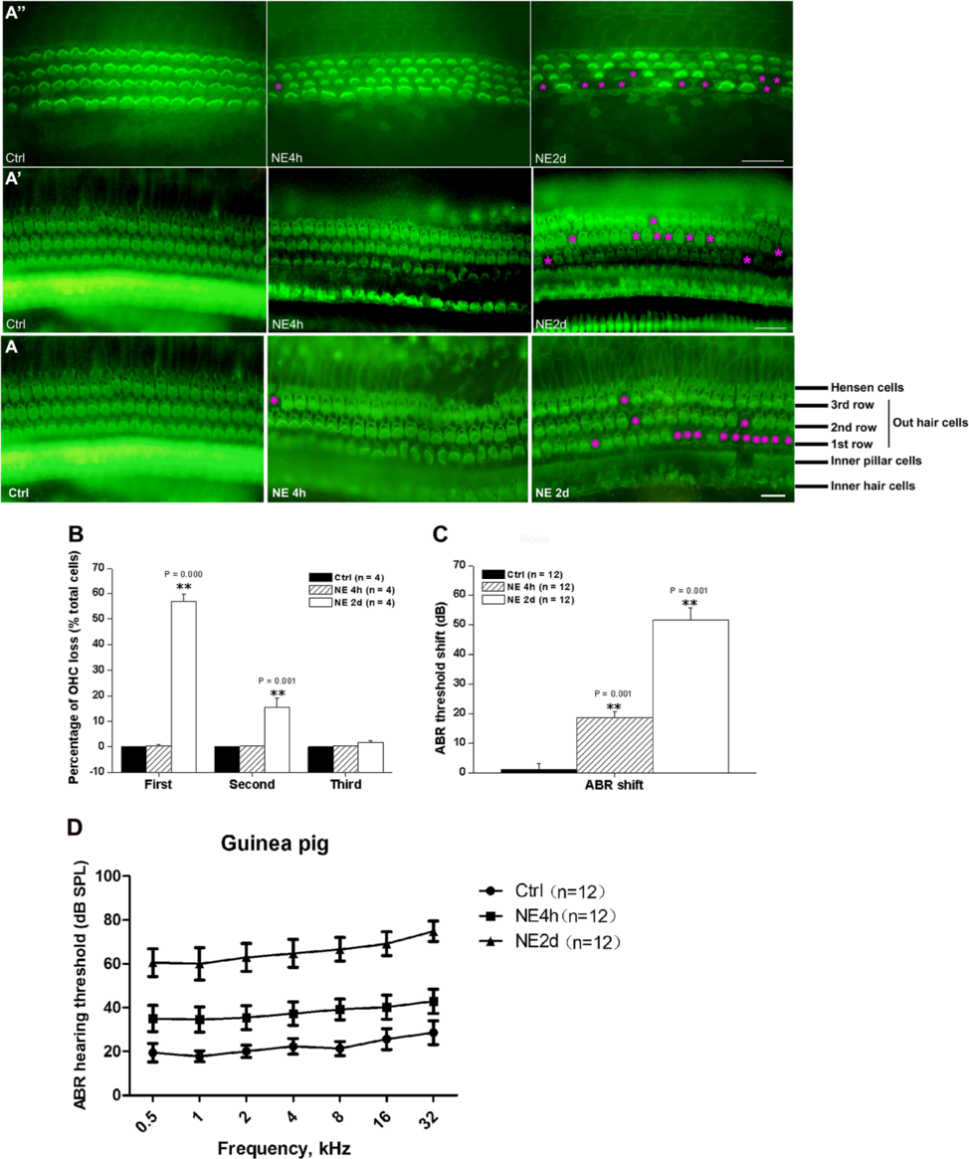
**Morphological alteration and functional impairment after NE. A)** Representative cochlear sensory epithelia surface preparation in the bottom basilar membrane structure from Ctrl, NE 4 h and NE 2d groups. **A’)** Morphological alteration of cochlear sensory epithelia surface preparation in the middle membrane structure from Ctrl, NE 4 h and NE 2d groups. **A”)** Morphological alteration of cochlear sensory epithelia surface preparation in the apex membrane structure from Ctrl, NE 4 h and NE 2d groups. **B)** Percentage of OHC loss in each row. The loss was observed mainly in the first row and the posterior second row. **C)** Mean ABR threshold shifts in Ctrl, NE 4 h and NE 2d groups. **D)** ABR hearing threshold to pure tone frequencies in Ctrl, NE 4 h and NE 2d groups. Scale bar = 20 μm in **A**, **A’** and **A”**. **P < 0.01, vs Ctrl, data were expressed as mean ± SEM.  and  indicate Hair Cell loss.

Cochlear surface preparation revealed a dose-dependent impairment to the basilar membrane structure as well as the apex, middle membrane structure. There was no loss of OHC in the control (Figure 1A, Ctrl) cochlear sample, few OHC loss in the NE 4 h sample (Figure 1A, NE 4 h) but severe loss of OHC in the NE 2d sample (Figure 1A, NE 2 h). Furthermore, the OHC loss was mainly involved in the first row of OHC. The outer hairs were well-organized in ‘V’ or ‘W’ shape in the Ctrl group, however, this organization was impaired in NE 4 h and NE 2d groups (Figure 1A). The morphology of inner hair cells (IHC) was not obviously altered by the NE but for the apex membrane structure of NE 2d group (Figure 1A’ and A”). And interestingly, after noise exposure, the lower part the membrane structure lied in, the greater damage it had (Figure 1A, A’ and A”).

One way ANOVA revealed a significant group difference for the OHC loss in the first (Group factor: NE, F(2,9) = 384.053, P = 0.000), second (Group factor: NE, F(2,9) = 18.454, P = 0.001) and third (Group factor: NE, F(2,9) = 4.300, P = 0.049) row (Figure 1B). The group difference mainly derived from the significant difference between Ctrl and NE 2d groups. *Post hoc* Turkey HSD test revealed that there was the significant difference between Ctrl and NE 2d group in the 1^st^ (P = 0.000, percentage of OHC loss = 0 ± 0% and 56.75 ± 2.35% for Ctrl and NE 2d, respectively), 2^nd^ (P = 0.001, percentage of OHC loss = 0 ± 0% and 15.50 ± 2.92% for Ctrl and NE 2d, respectively) but not the 3^rd^ (P = 0.057, percentage of OHC loss = 0 ± 0% and 1.75 ± 0.65% for Ctrl and NE 2d, respectively) row; however, no significant difference was found between Ctrl and NE 4 h group in the 1^st^ (P = 0.976), 2^nd^ (P = 0.996) or 3^rd^ (P = 0.921) row. Based on the above mentioned outer hair disorders, our data suggested that the NE for 4 h did impair the cochlear morphology but might not cause severe OHC loss.

We then expected the functional impairments after both NE 4 h and NE 2d and tested this expectation by measuring the ABR to both click stimuli and pure tone frequencies. The ABR threshold shifts to click stimuli were calculated by the post-threshold *minus* pre-threshold and one way ANOVA was performed to detect the significant change in the hearing function (Figure 1C). There was the significant group difference (Group factor: NE, F(2,33) = 80.464, P = 0.000) among 3 groups. *Post hoc* Turkey HSD test revealed that there was the significant difference between NE 2d (threshold shift for NE 2d = 51.500 ± 4.022 dB, P = 0.001) or NE 4 h (threshold shift for NE 4 h = 18.583 ± 1.987 dB, P = 0.001) and Ctrl (threshold shift for Ctrl = 1.167 ± 2.059 dB). Furthermore, NE 2d induced more severe hearing impairment than NE 4 h did (*Post hoc* Turkey HSD test, P = 0.001). In addition, the ABR hearing threshold to pure tone frequencies were also calculated by two-way ANOVA to detect the significant change in the hearing function (Figure 1D). There was also the significant group difference (Group factor: NE, Control factor: frequency, F(2,231) = 1484.20, P < 0.0001) among 3 groups.

All these data were for our concept that both NE 4 h and NE 2d induce hearing impairment and the morphological alterations of cochlea. We then asked how much the cochlear ultrastructure change can be caused by NE. Because the impairments to hearing and morphology were robust in both the NE 2d group and the NE 4 h group, we then compared the ultrastructure changes among Ctrl, NE 4 h and NE 2d groups.

### NE alters the cochlear ultrastructure and BLB permeability

**Figure 2:**
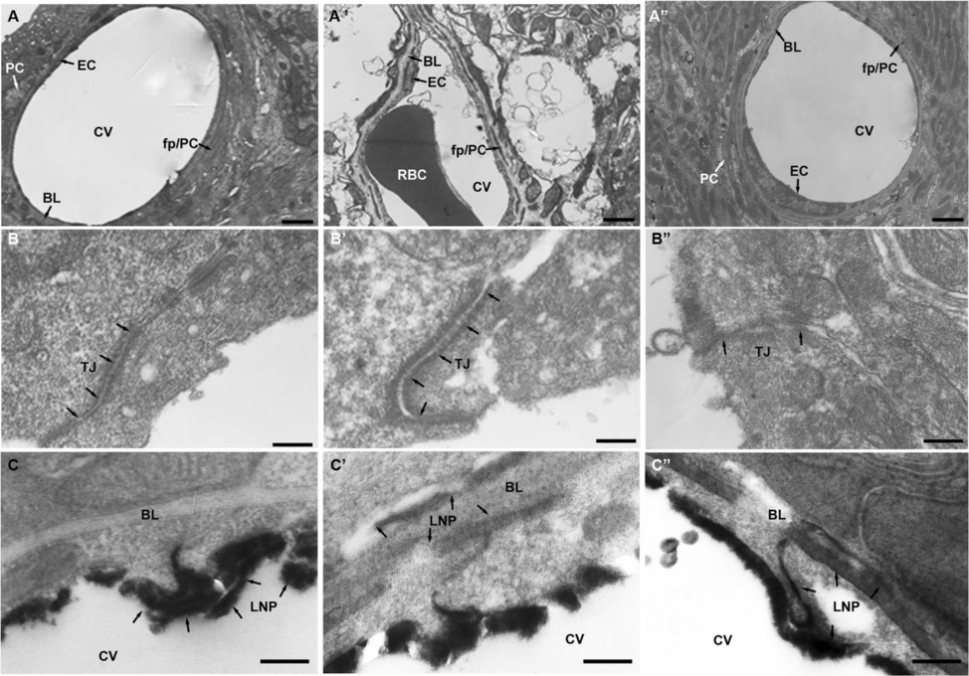
**The ultrastructure of cochlea from control (A,B), NE 2d (A’,B’) and NE 4 h (A”,B”) groups as well as the distribution of lanthanum nitrate particles from Control (C), NE 2d (C’) and NE 4 h (C”) groups examined with TEM. A)** Normal ultrastructure of BLB showing PC, EC and fp/PC. **B)** Normal ultrastructure of TJ. **C)** LNPs were restrained inside the cochlear vessels from a control animal. **A’**) Ultrastructure of BLB showing PC, EC and fp/PC after NE 2d. **B’**) Ultrastructure of TJ after NE 2d. **C’**) LNPs entered into the tissue after NE 2d. **A”**) Ultrastructure of BLB showing PC, EC and fp/PC after NE 4 h. **B”**) Ultrastructure of TJ after NE 4 h. **C”**) LNPs entered into the tissue after NE 4 h. Arrows in **B**, **B’ and B”** indicate TJ. BL, basal lamina; CV, capillary vessel; EC, endothelial cells; fp, pericyte foot; LNPs, lanthanum nitrate particles; PC, pericytes; RBC, red blood cells. Scale bar in **A**, **A’** and **A”** = 500 nm; in the other panels = 100 nm.

By using TEM, the changes in the integrity of TJs barrier in the stria vascularis after NE 2d or NE 4 h were observed. Pericytes (PC) in the stria vascularis of normal guniea pig were tightly associated with endothelial cells (EC), with foot processes (fp) positioned next to the endothelium (Figure 2A). A dense and continuous basal lamina (BL) is observed between pericytes and ECs (Figure 2A). Moreover, appearing as a series of electron-dense zones, TJ lay in the plasma membrane of adjacent ECs and sealed the intercellular cleft in normal cochlear microvessels (Figure 2B). In contrast, after NE 2d or NE 4 h, it was observed that PCs showed an abnormally loose association with ECs, with wide spaces separating some regions of the two types of cells and BL appeared less electron-dense (Figure 2A’ and 2A”). Also pericyte foot (pf) processes were migrating into the BL, and the size of pf processes was larger than that in a normal animal; meanwhile, the internal surface of vessel wall in a NE 2d or NE 4 h animal was not smoother than that in a normal one (Figure 2A’ and 2A”). TEM revealed the widening of TJ in the NE 2d or NE 4 h animal compared with normal animal (Figure 2B’ and 2B”).

We then investigated the potential permeability change of BLB after NE 2d or NE 4 h by observing the trace of LNPs in stria vascularis capillary under TEM. LNPs were restrained in the cochlear capillary vessel (CV) (Figure 2C) in the normal animal but entered into the tissue space via BLB after NE 2d or NE 4 h (Figure 2C’ and 2C”). All these data suggested that NE 2d did impair the cochlear ultrastructure, while NE 4 h partly damaged it and both of them increased the BLB permeability.

### NE alters the TJPs expression

We then asked what the involved molecules that contribute to the impairment of BLB integrity could be. Based on our hypothesis and previous reports, we targeted the TJ Proteins Claudin-5 and Occludin. Their expressions after NE 4 h or NE 2d were investigated with immunohistochemistry and western blot to reveal the potential involvements in the NE-induced BLB impairments.

**Figure 3:**
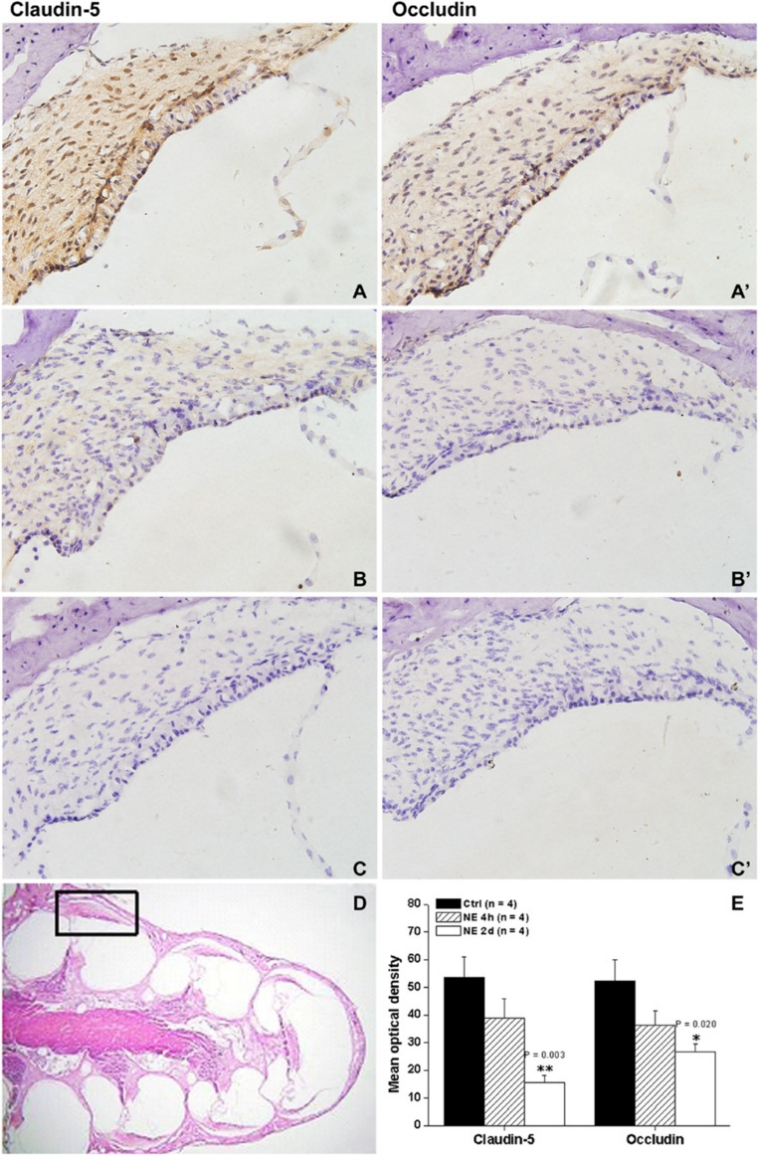
**The expressions of Claudin-5 (A,B,C) and Occludin (A’,B’,C’) in cochleae after NE 4 h or NE 2d were analyzed with immunohistochemistry.** **A)** Normal expression of Claudin-5 in cochlea. **A')** Normal expression of Occludin in cochlea. **B)** The decreased expression of Claudin-5 in cochlea after NE 4h. **B')** The decreased expression of Occludin in cochlea after NE 4h. **C)** The decreased expression of Claudin-5 in cochlea after NE 2d. **C')** The decreased expression of Occludin in cochlea after NE 2d. **D)** Normal structure of cochlea stained by HE. **E)** The expression of Claudin-5 and Occludin in cochleae were analyzed by optical density. The regions used for analysis was shown in **D**. The relative expression levels for Claudin-5 and Occludin were shown in **E**. Scale bar = 100 μm. *P < 0.05, **P < 0.05.

Immunohistochemistry analysis of Claudin-5 (Figure 3A,B,C) and Occludin (Figure 3A’,B’,C’) showed decreased cellular expression after both NE 4 h and NE 2d. One way ANOVA for Claudin-5 protein levels revealed a significant group difference (Group factor: NE, F(2,9) = 10.671, P = 0.004, Figure 3E). *Post hoc* Turkey HSD test revealed the significant difference between NE 2d (P = 0.003) but not NE 4 h (P = 0.234) and control. Furthermore, there was significant difference between NE 2d and NE 4 h groups (Turkey HSD test, P = 0.049). One way ANOVA for Occludin protein levels revealed a significant group difference (Group factor: NE, F(2,9) = 5.849, P = 0.024, Figure 3E). *Post hoc* Turkey HSD test revealed that the significant difference was between NE 2d (P = 0.020) but not NE 4 h (P = 0.141) and control. However, there was no difference between NE 2d and NE 4 h groups (Turkey HSD test, P = 0.447).

**Figure 4:**
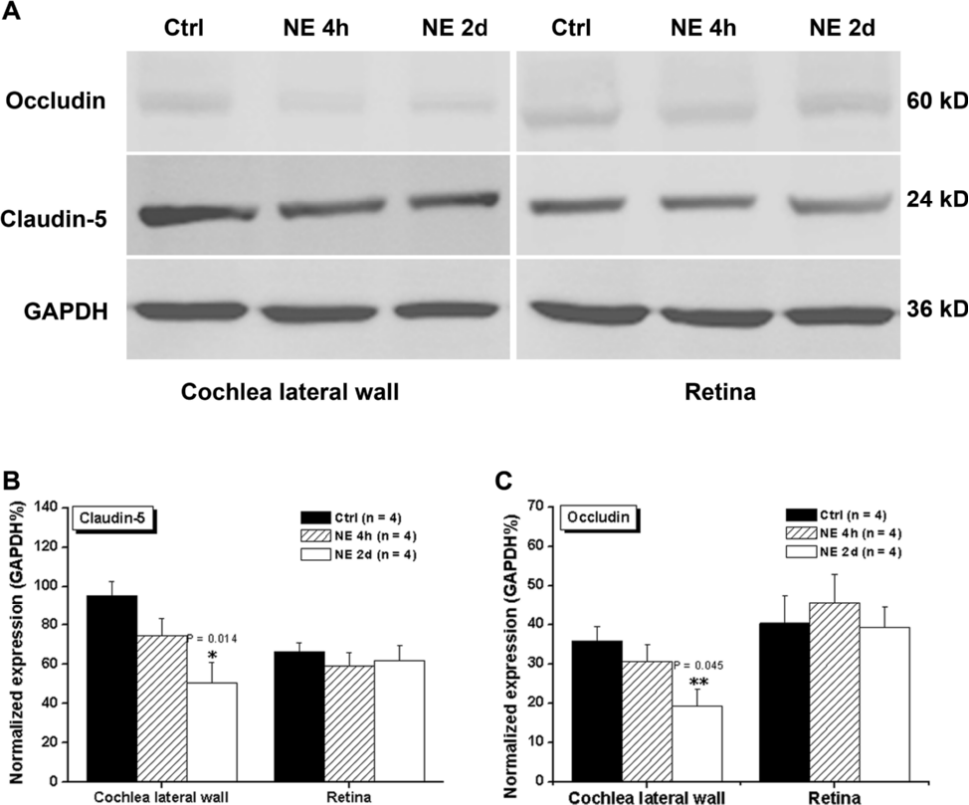
**The protein expressions of Claudin-5 (A,B) and Occludin (A,C) in cochlear after NE 4 h or NE 2d were analyzed with western blot.** The protein expressions in retina were utilized for negtive control. The intensities of GAPDH bands were used for normalization. *P < 0.05, **P < 0.05.

To confirm the effect of NE on the expression levels of Claudin-5 and Occludin, we detected their protein levels with western blot and the specificity of NE effect was tested by adding retina tissue as control (Figure 4A). One way ANOVA for Claudin-5 protein in cochlear revealed a significant group difference (Group factor: NE, F(2,9) = 6.618, P = 0.017, Figure 4B). Post *hoc* LSD test revealed the significant difference between NE 2d (P = 0.014) but not NE 4 h (P = 0.265) and control. However, there was no difference between NE 4 h and NE 2d groups (Turkey HSD test, P = 0.179). One way ANOVA for Occludin protein in cochlear revealed a significant group difference (Group factor: NE, F(2,9) = 4.295, P = 0.049, Figure 4C). *Post hoc* Turkey HSD test revealed that the significant difference was between NE 2d (P = 0.045) but not NE 4 h (P = 0.651) and control. However, there was no difference between NE 2d and NE 4 h groups (Turkey HSD test, P = 0.177).

## Discussion

In the current study, we confirmed that NE could induce significant hearing impairment and NE 2d contributed to significant OHC loss as well as ultrastructure changes including loosen TJ and increased permeability of BLB. Furthermore, these functional and ultrastructure changes were accompanied with significant down-regulation of TJ proteins Claudin-5 and Occludin. All these findings suggest that Claudin-5 and Occludin are involved in the noise-induced BLB ultrastructure changes, increased BLB permeability and the hearing impairment.

### NE impairs cochlear ultrastructure and BLB permeability

It is commonly accepted that NE causes significant hearing impairment [4,20] and corresponding OHC loss [17]. However, there is not a systematic observation at EM level to reveal the BLB ultrastructure changes that are very important for the hearing function. In one previous study, scan electronmicroscope (SEM) was used to reveal the surface change of BLB but not the underlying ultrastructure changes [17]. In another report, TEM was used to reveal the pericyte responses after NE but not the TJ structure [21]. We moved forward to investigate the BLB ultrastructure changes by using TEM and it revealed the featured loosen TJ just like those observed in the impaired blood–tumor barrier [22] or BBB [13]. Other finding of loosen TJ is consistent with a recent report on the noise-induced BLB ultrastructure change [20]. All these findings offered morphological evidences for the nosie-induced hearing impairment.

A suggested underlying mechanism for noise-induced hearing impairment is the increased BLB permeability. However, whether this is true remains inconclusive because there is the report that is against this concept [10]. Thus, we further investigated this issue by using LNP tracing technique and we confirmed the increased permeability after NE 2d or NE 4 h under TEM. Our conclusion is consistent with most previous reports where different tracing techniques were used [6,23]. The reason for the discrepancy of these findings may be that the different NE delivery protocols were used in different study. A relative acute NE was used in the study of Laurell et al. and the altered BLB permeability was not observed, while a relatively chronic or severe NE (3 h/d for 2 d in Suzuki et al.; 3 h/d for 7 d in Konishi et al. and 6 h/d for 2d in ours) was accompanied with the BLB permeability increase. To confirm whether the increased BLB permeability is the underlying reason, future study of BLB permeability after acute NE is yet to be explored.

### NE impairment accompanied with the TJP alterations

It is an interesting and challenging question that what is the underlying molecular events responsible for the noise-induced BLB permeability increase. Because of the similarity among BLB, BBB and blood-CSF barrier, etc. [24], we screened potential molecules based on the BBB studies and focused on TJPs Claudin-5 and Occludin that contribute to the integrity of BBB [15]. Occludin is expressed in cochlea but there is no report on the cochlear expression of Claudin-5 [20]. We first confirmed that both Occludin and Claudin-5 showed cellular expression within cochlea and then demonstrated a specific cochlea “dose-dependent” decrease that responded to NE. All these findings suggest that Claudin-5 and Occludin expression changes after NE contribute to the TJ ultrastructure alteration which finally leads to the increase of BLB permeability.

### Clinical implications of the study

In the current study, we provided evidence that NE induces OHC loss, cochlea TJ loosen and BLB permeability impairment. Furthermore, we revealed a specific decrease of TJPs Claudin-5 and Occlud in cochlear. All our findings suggest that there is a close relationship between TJPs and BLB integrity as well as the hearing function. Based on these findings and potential causal relationship between molecular event and the behavioral phenotype, and combined with the future cochlear target drug delivering system, BLB permeability drugs targeting TJPs may be found in their clinic application, although there is still a long journey for scientists to go.

### Limitations of this study

Our study has several limitations. First, we are not clear about the causal relationship between these molecular events and the ultrastructure changes, BLB permeability increase as well as the hearing impairments after NE. This issue might be resolved by focusing more on reversing TJP decrease with application of agonist, inhibitor or plasmids *in vivo*, and detecting whether counteracting TJPs’ decrease can protect the cochlear ultrastructure and BLB permeability as well as the hearing impairment after NE. However, due to the agonist specificity as well as the technique difficulties and limitations, we could not completely perform these experiments at the current stage and further efforts are needed to answer this question. Other limitations of this study are, second, whether the morphological change of cochlea is reversible or not; Third, whether chronic NE induces the same ultrastructure change or not. We are currently working on resolving these issues for our next project.

## Conclusions

Noise exposure, which induced significant hearing impairment and outer hair cell (OHC) loss, demolished tight junction and increased the permeability of BLB along with a dose-dependent decrease of Claudin-5 and Occludin expression. These data suggest that Claudin-5 and Occludin are closely related with the noise-induced BLB ultrastructure changes, increased BLB permeability and the hearing impairment, but the causal relationship needs to be further investigated.

## Abbreviations

ABR: Auditory brainstem responses
OHC: Outer hair cell
BLB: Blood-labyrinth barrier
TEM: Transmission electron microscope
NE: Noise exposure
TJP: Tight junction proteins

## References

[1] Lim DJ (1986). Effects of noise and ototoxic drugs at the cellular level in the cochlea: a review. Am J Otolaryngol.

[2] Fridberger A, Flock A, Ulfendahl M, Flock B (1998). Acoustic overstimulation increases outer hair cell Ca2+ concentrations and causes dynamic contractions of the hearing organ. Proc Natl Acad Sci U S A.

[3] Henderson DHB, Bielefeld EC, Schacht JPA, Fay RR (2008). Patterns and mechanisms of noise induced cochlear pathology. Auditory trauma, protection, and repair.

[4] Goldwyn BG, Quirk WS (1997). Calcium channel blockade reduces noise-induced vascular permeability in cochlear stria vascularis. Laryngoscope.

[5] Ohlemiller KK, Wright JS, Dugan LL (1999). Early elevation of cochlear reactive oxygen species following noise exposure. Audiol Neurootol.

[6] Suzuki M, Yamasoba T, Ishibashi T, Miller JM, Kaga K (2002). Effect of noise exposure on blood-labyrinth barrier in guinea pigs. Hear Res.

[7] Juhn SK, Hunter BA, Odland RM (2001). Blood-labyrinth barrier and fluid dynamics of the inner ear. Int Tinnitus J.

[8] Juhn SK, Rybak LP (1981). Labyrinthine barriers and cochlear homeostasis. Acta Otolaryngol.

[9] Yang Y, Dai M, Wilson TM, Omelchenko I, Klimek JE, Wilmarth PA, David LL, Nuttall AL, Gillespie PG, Shi X (2011). Na+/K + −ATPase alpha1 identified as an abundant protein in the blood-labyrinth barrier that plays an essential role in the barrier integrity. PLoS One.

[10] Laurell GF, Teixeira M, Duan M, Sterkers O, Ferrary E (2008). Intact blood-perilymph barrier in the rat after impulse noise trauma. Acta Otolaryngol.

[11] Liu X, Zheng G, Wu Y, Shen X, Jing J, Yu T, Song H, Chen J, Luo W (2013). Lead exposure results in hearing loss and disruption of the cochlear blood-labyrinth barrier and the protective role of iron supplement. Neurotoxicology.

[12] Liu J, Jin X, Liu KJ, Liu W (2012). Matrix metalloproteinase-2-mediated occludin degradation and caveolin-1-mediated claudin-5 redistribution contribute to blood–brain barrier damage in early ischemic stroke stage. J Neurosci.

[13] Jiao H, Wang Z, Liu Y, Wang P, Xue Y (2011). Specific role of tight junction proteins claudin-5, occludin, and ZO-1 of the blood–brain barrier in a focal cerebral ischemic insult. J Mol Neurosci.

[14] Balbuena P, Li W, Ehrich M (2011). Assessments of tight junction proteins occludin, claudin 5 and scaffold proteins ZO1 and ZO2 in endothelial cells of the rat blood–brain barrier: cellular responses to neurotoxicants malathion and lead acetate. Neurotoxicology.

[15] Nag S, Venugopalan R, Stewart DJ (2007). Increased caveolin-1 expression precedes decreased expression of occludin and claudin-5 during blood–brain barrier breakdown. Acta Neuropathol.

[16] Itoh M, Furuse M, Morita K, Kubota K, Saitou M, Tsukita S (1999). Direct binding of three tight junction-associated MAGUKs, ZO-1, ZO-2, and ZO-3, with the COOH termini of claudins. J Cell Biol.

[17] Li X, Mao XB, Hei RY, Zhang ZB, Wen LT, Zhang PZ, Qiu JH, Qiao L (2011). Protective role of hydrogen sulfide against noise-induced cochlear damage: a chronic intracochlear infusion model. PLoS One.

[18] Hoffstein S, Gennaro DE, Fox AC, Hirsch J, Streuli F, Weissmann G (1975). Colloidal lanthanum as a marker for impaired plasma membrane permeability in ischemic dog myocardium. Am J Pathol.

[19] Leeson TS, Higgs GW (1982). Lanthanum as an intracellular stain for electron microscopy. Histochem J.

[20] Yang Y, Yang Y, Teresa M, Wilson TM, Omelchenko I, John E, Klimek JE, Phillip A, Wilmarth PA, Larry L, David LL, Alfred L, Nuttall AL, Peter G, Gillespie PG, Shi X (2011). Na+/K + −ATPase a1 Identified as an Abundant Protein in the Blood-Labyrinth Barrier That Plays an Essential Role in the Barrier Integrity. PLoS One.

[21] Shi X (2009). Cochlear pericyte responses to acoustic trauma and the involvement of hypoxia-inducible factor-1alpha and vascular endothelial growth factor. Am J Pathol.

[22] Liu LB, Xue YX, Liu YH, Wang YB (2008). Bradykinin increases blood-tumor barrier permeability by down-regulating the expression levels of ZO-1, occludin, and claudin-5 and rearranging actin cytoskeleton. J Neurosci Res.

[23] Konishi T, Salt AN, Hamrick PE (1982). Effects of exposure to noise on permeability to potassium of the endolymph-perilymph barrier in guinea pigs. Acta Otolaryngol.

[24] Inamura N, Salt AN (1992). Permeability changes of the blood-labyrinth barrier measured in vivo during experimental treatments. Hear Res.

